# Routine laboratory biomarker signature combined with machine learning for rapid differentiation of intracerebral hemorrhage from acute ischemic stroke

**DOI:** 10.3389/fneur.2026.1931764

**Published:** 2026-10-15

**Authors:** Xiaoyan Hao, Juan Wang, Jing Wang, Yujiao Hu, Fengjuan Wang, Xinran Liu, Congxia Bai

**Affiliations:** Department of Clinical Laboratory Medicine, Xijing Hospital, Fourth Military Medical University, Xi'an, China

**Keywords:** biomarker validation, clinical diagnostics, intracerebral hemorrhage, ischemic stroke, machine learning, routine laboratory indicators

## Abstract

**Background:**

Rapid and reliable differentiation between intracerebral hemorrhage (ICH) and acute ischemic stroke (IS) is essential for appropriate triage and treatment. We aimed to develop and validate a laboratory-based machine learning model using routine clinical chemistry and hematology parameters to discriminate ICH from IS.

**Methods:**

We retrospectively analyzed the clinical and laboratory data of 12,213 hospitalized stroke patients (including 3,251 with ICH and 8,962 with IS) and 2,893 hypertensive controls at Xijing Hospital between 2013 and 2023. A temporal validation cohort comprising 153 ICH and 342 IS patients admitted from January to December 2024 was also established. To address class imbalance, BorderlineSMOTE-2 (BS2) was applied. Candidate variables were screened using three feature selection strategies, including RFECV-ADA, Lasso, and Boruta. Six supervised machine learning models were trained using ten-fold cross-validation. Model performance was evaluated by the area under the receiver operating characteristic curve (AUC), sensitivity, specificity, and accuracy. Feature importance was interpreted using SHAP analysis. Mendelian randomization and colocalization analyses were performed as exploratory complementary analyses.

**Results:**

Ten routine laboratory parameters were retained for model construction: white blood cell count, neutrophil percentage, cystatin C, uric acid, total protein, potassium, sodium, chloride, fibrinogen degradation product, and D-Dimer. In the test cohort, the final model (BS2_LightGBM_V10) achieved AUCs of 0.926 for discriminating ICH from IS, 0.979 for discriminating ICH from controls, and 0.923 for discriminating IS from controls. For the three-class classification task (ICH, IS, and controls), the overall accuracy reached 79.07%. SHAP analysis indicated that neutrophil percentage, cystatin C, D-Dimer, and total protein were the most influential predictors. Mendelian randomization analysis suggested that uric acid had a potential causal association with the risk of ICH (OR = 1.309), whereas uric acid (OR = 1.0008), cystatin C (OR = 1.0011), and potassium (OR = 1.0040) had potential causal associations with the risk of IS. Colocalization analysis identified 18 genes linked to uric acid in the context of ICH.

**Conclusion:**

A diagnostic model based on ten routine laboratory indicators accurately differentiated ICH from IS. Uric acid may also play a causal role in the pathogenesis of both disorders.

## Introduction

Stroke is the leading cause of death in China and the second leading cause of death worldwide, posing a significant threat to global population health (1). There are more than 100 million stroke patients and 12 million new stroke cases reported globally each year, with almost 7 million deaths, and stroke is the third leading cause of disability (2). Ischemic stroke (IS) accounting for 65.3% of incident strokes, whereas intracerebral hemorrhage (ICH) accounts for approximately 28.8% of all strokes in China and has an exceedingly high mortality rate of 67.9% (3). Hypertension is recognized as a significant risk factor for stroke, accounting for approximately 65% of ICH cases; it induces chronic arterial damage and significantly amplifies adverse cerebrovascular outcomes (4).

Given the fundamental differences in pathophysiological mechanisms between ICH and IS, these conditions exhibit substantial heterogeneity in their clinical manifestations, therapeutic strategies, preventive measures, and epidemiological characteristics. Given the fundamental differences in pathophysiological mechanisms between ICH and IS, these conditions exhibit substantial heterogeneity in their clinical manifestations, therapeutic strategies, preventive measures, and epidemiological characteristics. Although non-contrast CT can currently differentiate ICH from IS rapidly with relatively high sensitivity, its clinical application is severely constrained by the high cost of equipment and the reliance on professional radiographers and interpreting physicians. Therefore, it is necessary to develop a predictive model based on routinely available blood biomarkers and clinical parameters that does not depend on large-scale imaging equipment (5).

Machine learning technologies have recently inspired new research directions in the fields of disease prediction, diagnosis, and treatment strategy optimization. Machine learning algorithms can efficiently extract underlying patterns and associations from heterogeneous multisource data, including vast clinical records, high-dimensional imaging data, and clinical laboratory biomarkers (6). However, in clinical practice, clinicians tend to focus on markedly abnormal values and may miss finer patterns and correlations among laboratory measures, which leads to underutilization of the diagnostic capacity of laboratory tests. Consequently, an integrated interpretation of clinical laboratory tests is essential for facilitating early disease diagnosis and identifying preventable risk factors. Machine learning-based stroke risk prediction models have been developed using clinical and imaging data, and neuroimaging techniques have been applied to distinguish stroke subtypes (5, 7–9). Researchers have developed machine learning models using clinical data to predict functional outcomes in acute IS and have identified blood-based biomarkers for IS prognosis (10, 11). Furthermore, Mendelian randomization (MR) analyses have revealed potential causal relationships between these biomarkers and subtypes of IS from a genetic epidemiological perspective (12). Despite these advances, a rapid, cost-effective prediction model to distinguish between ICH and IS based on routine laboratory indicators is lacking.

Therefore, this study aims to develop a machine learning-based predictive model using routinely accessible laboratory indicators to discriminate stroke subtypes. In addition, we screened genetic instruments related to candidate indicators and combined them with genome-wide association study data for ICH and IS to conduct MR analysis. Positive results from the MR analysis were then subjected to colocalization analysis to explore the potential mechanisms by which these indicators influence the risk of ICH and IS (Figure 1).

**Figure 1:**
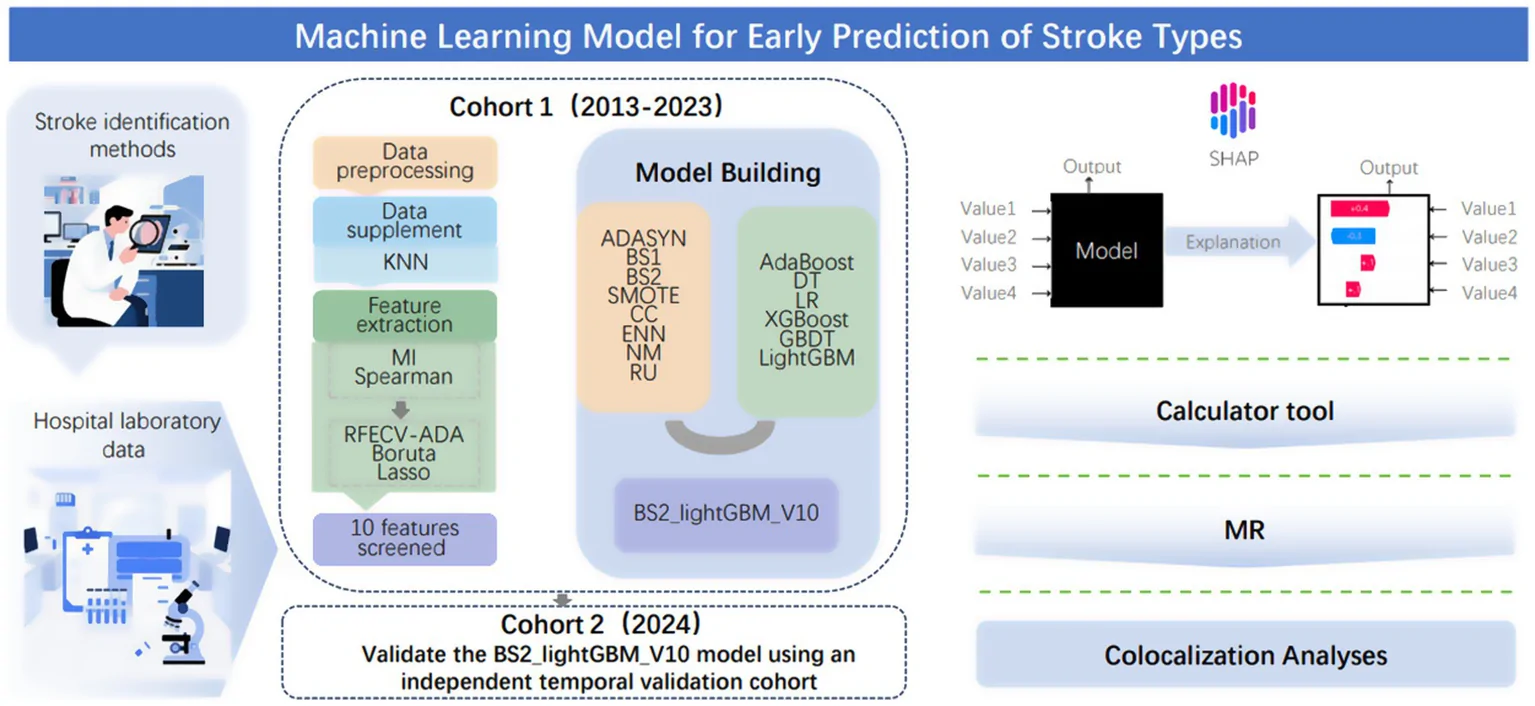
Flow chart of this study. MI, mutual information; RU, RandomUnderSampler; NM, NearMiss; CC, ClusterCentroids; ENN, EditedNearestNeighbours; BS1/2, BorderlineSMOTE1/2; SMOTE, synthetic minority oversampling technique; DT, decision tree; LR, logistic regression; XGBoost, eXtreme Gradient Boosting; GBDT, gradient boosting decision tree; LightGBM, light gradient boosting machine; MR, Mendelian randomization.

## Methods and materials

### Study design and participants

We retrospectively collected data from 3,251 ICH patients and 8,962 IS patients admitted to the Department of Neurology and Neurosurgery at Xijing Hospital between January 2013 and December 2023 and enrolled them in the study cohort (cohort 1). Additionally, 2,893 individuals with simple hypertension were enrolled as controls. As a temporal validation cohort (cohort 2), 153 ICH patients and 342 IS patients admitted to the same department between January 2024 and December 2024 were included. All data preprocessing and training-related operations during the model development phase, including normalization, missing value imputation (KNN), feature engineering, hyperparameter optimization, cross-validation, and resampling, were strictly performed within Cohort 1. Cohort 2, serving as an independent temporal validation set, was not involved in any part of the model construction process. The inclusion criteria were as follows: (1) age >18 years; (2) meeting the diagnostic criteria for ICH and IS established by the Cerebrovascular Disease Group of the Chinese Academy of Neurological Sciences; and (3) exclusion of secondary causes of ICH, such as trauma, hemorrhagic transformation of IS, cerebrovascular malformations, or brain tumors. The demographic and clinical characteristics of the study population were obtained by consulting hospital records or medical examination records, and laboratory indicators were obtained by consulting the LIS system. For all the patients, clinical laboratory test data (including routine blood test results and blood biochemical and coagulation indicators) were collected from the first test after admission. Following the exclusion of features with >30% missing data, significance testing yielded 47 features. This study was approved by the Review Committee of Xijing Hospital (Approval No. KY20242401-F-1).

### Double-stage feature screening strategy

To identify biomarkers for distinguishing stroke subtypes, we employed a two-stage progressive feature screening strategy designed to balance feature selection efficiency with model stability. In the first stage, coarse screening was implemented using a statistical method (13, 14): mutual information (MI) was computed to quantify feature–target variable associations (Equations 1, 2), while Spearman’s rank correlation coefficient was simultaneously used to assess the monotonic correlations between features. By retaining the top 30 features from each method, 25 features were found to be common to both, thereby effectively eliminating those with insufficient information content. Three machine learning algorithms (4, 15) were integrated in the second stage for refined screening: an RFECV-ADA (AdaBoost-based Recursive Feature Elimination with Cross-Validation) framework was constructed to iteratively optimize the feature subset through cross-validation; the Boruta feature selection algorithm, which performs 1,000 random-forest importance permutation tests via shadow features to retain only features whose importance significantly exceeds random noise (*p* < 0.01), was applied; and Lasso (least absolute shrinkage and selection operator) regularization was employed for sparse constraint optimization, with parameters optimized using the coordinate descent algorithm. The final set of core features was determined on the basis of the consensus among the three feature selection methods in the second stage, yielding a total of 10 features: white blood cell count (WBC), neutrophil percentage (NEUT%), cystatin C (CysC) levels, uric acid (UA) levels, total protein (TP), potassium (K^+^) levels, sodium (Na^+^) levels, chloride (Cl^−^) levels, fibrinogen degradation product (FDP) and D-Dimer levels for subsequent analysis.

The MI between discrete random variables X and Y can be calculated as follows:


$$I(X;Y)=\sum_{y\in Y}\sum_{x\in X}p(x,y)\log(\frac{p(x,y)}{p(x)p(y)})$$ (1)

where *p*(*x*,*y*) represents the joint probability mass function of X and Y and *p*(*x*) and *p*(*y*) denote the marginal probability mass functions of *X* and *Y*, respectively.

The MI between continuous random variables X and Y can be calculated as follows:


$$I(X;Y)=\int \int p(x,y)\log(\frac{p(x,y)}{p(x)p(y)})\text{dxdy}$$ (2)

where *p*(*x*,*y*) represents the joint probability mass function of *X* and *Y*, while *p*(*x*) and *p*(*y*) denote the marginal probability mass functions of *X* and *Y*, respectively.

### Resampling techniques

To effectively mitigate the adverse impact of prevalent intergroup data imbalance in ML classification tasks on model performance, this study systematically implemented multiple resampling techniques for data preprocessing using Python’s imblearn package. In terms of methodological design, this research employed complementary undersampling and oversampling strategies (16–19), encompassing eight representative resampling algorithms, to comprehensively evaluate their performance optimization effects on the model.

To address the class imbalance issue, we employed undersampling techniques to reduce the number of IS cases, thereby matching the sample size of the ICH group. Four representative undersampling techniques were employed: RU (RandomUnderSampler), which achieves balance by randomly removing majority-class samples, offering operational simplicity but risking information loss; NM (NearMiss), which preserves proximity samples based on distance metrics to optimize data distribution; CC (ClusterCentroids), which uses K-means to generate cluster centroids as substitutes for majority-class samples; and ENN (EditedNearestNeighbours), which enhances decision accuracy by eliminating boundary noise through KNN. Similarly, the number of ICH cases was increased to match that of IS using an oversampling technique. Four oversampling algorithms were implemented: ADASYN, which adaptively focuses on difficult-to-classify regions for sample generation; BS1/2 (BorderlineSMOTE1/2), which performs single- and dual-directional boundary sample interpolation; and the classic SMOTE (synthetic minority oversampling technique), which synthesizes samples via linear interpolation among K-nearest neighbors (neighbors = 5). By systematically integrating undersampling and oversampling methods, this study established a comprehensive experimental framework, thus providing the data foundation for model comparison.

### Final model establishment

By employing the Python scikit-learn framework and the ten selected features, prediction models were constructed using six ML algorithms: AdaBoost (adaptive boosting), DT (decision tree), LR (logistic regression), XGBoost (eXtreme Gradient Boosting), GBDT (gradient boosting decision tree), and LightGBM (light gradient boosting machine) (20–24). Models were constructed on Cohort 1 using 10-fold cross-validation with StratifiedKFold stratification to ensure that the class proportions in each fold were consistent with those in the original dataset. A combined strategy of randomized grid search and cross-validation was employed for hyperparameter optimization (25). Model performance was evaluated using SEN (sensitivity), SPE (specificity), ACC (accuracy), PPV (positive predictive value), NPV (negative predictive value), F1 score, and AUC (area under the curve) (with 95% CI). The model adopted 0.5 as the probability threshold for classification decisions. The 95% confidence intervals of the model AUCs were calculated using the Hanley–McNeil method based on the normal approximation. The algorithm execution time was also measured using Python’s time module to assess computational efficiency. SHAPs (SHapley Additive exPlanations) were used to show the contribution of individual variables to each category via the shapviz R package (22).

### PyQt5-based GUI construction

In this study, a GUI (graphical user interface) application was developed using Python’s built-in PyQt5 framework (26). The interface design incorporates data input controls, parameter configuration panels, and visualization modules for result display, employing a MVC (Model–View–Controller) architecture to ensure functional decoupling and extensibility. The core computational module integrates the optimized BS2_LightGBM_V10 prediction model with fine-tuned hyperparameters.

### MR analysis of source data

To further investigate whether the aforementioned nine indicators are causally related to the occurrence of ICH and IS, we further assessed GWAS summary data of plasma WBC, NEUT%, CysC, UA, TP, K^+^, Na^+^, Cl^−^ and D-Dimer levels from the IEU Open GWAS database (https://gwas.mrcieu.ac.uk/, Supplementary Table 1). We selected SNPs associated with the exposure that reached genome-wide significance at *p* < 5 × 10^−8^ (WBC, NEUT%, CysC, UA, TP, Na^+^, Cl^−^), *p* < 5 × 10^−6^(K^+^, D-Dimer)as IVs. Regulatory regions were defined within 10,000 kilobases on both sides of the coding sequence, and linkage disequilibrium clustering was performed with R^2^ < 0.001. The F statistic was calculated to evaluate the strength of the association between the IVs and the exposure factor using the formula F = (beta/se)^2^ (27), where beta represents the allelic effect size and SE denotes the standard error. SNPs with *F* values of less than 10 were excluded to prevent weak IV bias.

The outcome data were sourced from the IEU Open GWAS database, the ICH phenotypes dataset included 1,935 cases and 471,578 controls of European ancestry, involving 24,191,284 SNPs,^1^ and the IS phenotype dataset included 1,420 cases and 461,590 controls of European ancestry, involving 9,851,867 SNPs.^2^ We harmonized the IVs with the outcome data.

### MR analysis

Two-sample MR analyses were conducted using the TwoSampleMR package (version 0.6.2) to investigate the causal effects between the indicators and the risk of ICH and IS (28). To assess the genetic correlation between exposure and outcome data, this study employed LDSC (linkage disequilibrium score regression) (29) to estimate the covariance attributable to sample overlap on the basis of Z scores from a GWAS of the exposure and outcome. We employed five MR methods, including IVW (inverse variance weighting), MR-Egger regression, the weighted median approach, simple mode, and weighted mode (30, 31), to estimate the causal effects of genes on ICH and IS. The IVW method served as the main foundation for the results of this analysis, whereas the other methods served as supplementary analyses (32). After MR positive results were obtained, we used MR-Egger and IVW statistical tests to assess robustness (33). The Cochran Q statistic was used to detect heterogeneity in the results, with a significance level of *p* < 0.05 indicating the presence of heterogeneity. MR-Egger and MR-PRESSO (MR pleiotropy residual sum) and outlier were used to assess horizontal pleiotropy, with *p* < 0.05 suggesting the presence of horizontal pleiotropy (34).

### Colocalization analysis

Following the completion of the 2-sample MR analysis, we identified the exposure with positive results (UA, CysC and K^+^) and conducted colocalization analysis with ICH and IS. We used the coloc package^3^ (35) to assess whether the exposure and outcome were linked to the same genomic region. Our criterion for colocalization required a PPH4 > 80%, indicating that genes in that region were associated with both the exposure and the outcome (36). Finally, we used the gassocplot2 package to visualize the colocalization results, providing a clear display of the genes associated with UA in relation to ICH.

### Statistical analysis

Statistical analysis was performed using SPSS 23.0 (IBM Corp., NY, USA). The sample distribution was determined using the Kolmogorov–Smirnov normality test. For parametric data, the 2-tailed unpaired Student’s t test was used to determine differences between 2 groups. One-way analysis of variance and the Bonferroni *post hoc* correction were used to evaluate >2 groups. For nonparametric data, the Mann–Whitney *U* test with the exact method was used to identify differences between 2 groups. Statistical comparisons for percentages were performed using chi-square statistical analysis. Data are presented as the mean ± SD or median and interquartile range (IQR) [M(IQR)]. *p* < 0.05 was considered to indicate statistical significance.

## Results

### Clinical characteristics of the participants

A total of 3,251 patients with ICH and 8,962 with IS were enrolled in the first phase of the study. The baseline clinical characteristics and laboratory indicators of these ICH and IS patients are summarized (Table 1). Laboratory indicators were included only if the rate of missing data was strictly less than 30%, ensuring analytical representativeness and reliability. Ultimately, 47 laboratory indicators (including routine blood test results, blood biochemistry, coagulation profiles, electrolytes, etc.) were included, with significant differences observed between ICH and IS. Specifically, the WBC was significantly greater in the ICH group than in the IS group (9 (6.52, 12.34) vs. 6.52 (5.25, 8.2); *p* < 0.001). Similarly, NEUT% was markedly greater in ICH patients (0.783 (0.654, 0.868) vs. 0.642 (0.571, 0.724); *p* < 0.001). Furthermore, patients with IS had lower TP levels than ICH patients did. Indicators reflecting glomerular filtration function, namely, UA and CysC, were significantly elevated in the IS group. The levels of K^+^, Na^+^, and Cl^−^ ions generally increased in IS patients, suggesting an imbalance in electrolyte homeostasis. Conversely, FDP and D-Dimer levels tended to decrease in the IS group.

**Table 1:** Demographics and clinical characteristics.

Variable	ICH (*n* = 3,251)	IS (*n* = 8,962)	*p* value
Age, years	58 (49, 67)	63 (54, 72)	<0.001
Sex			<0.001
Male	2,148	6,246	
Female	1,103	2,716	
Neutrophil Ratio (NEUT%), %	0.783 (0.654, 0.868)	0.642 (0.571, 0.724)	<0.001
Lymphocyte Ratio (LYMPH%), %	0.141 (0.076, 0.248)	0.261 (0.187, 0.325)	<0.001
Monocyte Ratio, %	0.059 (0.044, 0.075)	0.066 (0.054, 0.08)	<0.001
Eosinophil Ratio, %	0.005 (0, 0.016)	0.016 (0.008, 0.028)	<0.001
Basophil Ratio, %	0.002 (0.001, 0.004)	0.003 (0.002, 0.005)	<0.001
Neutrophil Count (NEUT#), ×10^9^/L	6.93 (4.29, 10.28)	4.11 (3.09, 5.66)	<0.001
Lymphocyte Count (LYMPH#), ×10^9^/L	1.23 (0.81, 1.76)	1.59 (1.21, 2.03)	<0.001
Monocyte Count, ×10^9^/L	0.5 (0.35, 0.7)	0.43 (0.33, 0.56)	<0.001
Eosinophil Count, ×10^9^/L	0.04 (0, 0.11)	0.1 (0.05, 0.17)	<0.001
Basophil Count, ×10^9^/L	0.02 (0.01, 0.03)	0.02 (0.01, 0.03)	0.777
Red Blood Cell Count (RBC), ×10^12^/L	4.48 (4.03, 4.91)	4.52 (4.14, 4.9)	0.001
Hemoglobin (HGB), g/L	138 (125, 151)	139 (128, 151)	0.002
Hematocrit (HCT)	0.41 (0.374, 0.444)	0.416 (0.384, 0.447)	<0.001
Mean Corpuscular Volume (MCV), fL	91.4(88.4, 94.8)	92.2(89, 95.3)	<0.001
Mean Corpuscular Hemoglobin (MCH), pg	30.8 (29.7, 32)	30.8 (29.8, 31.9)	0.853
Mean corpuscular hemoglobin concentration (MCHC), g/L	336 (328, 345)	334 (326, 342)	<0.001
Red Cell Distribution Width Coefficient of Variation (RDW-CV)	0.13 (0.125, 0.137)	0.13 (0.125, 0.136)	0.131
Red Cell Distribution Width Standard Deviation (RDW-SD), fL	43.3 (41.1, 46.3)	43.6 (41.3, 46.2)	0.003
Platelet count (PLT), ×10^9^/L	194 (152, 242)	195 (158, 238)	0.214
Platelet Distribution Width (PDW), fL	13 (11.4, 15.45)	13.3 (11.7, 15.6)	<0.001
Mean Platelet Volume (MPV), fL	10.9 (10.1, 11.8)	11 (10.2, 11.8)	0.01
Platelet-Larger Cell Ratio (P-LCR), %	0.317 (0.254, 0.392)	0.326 (0.264, 0.397)	0.001
White Blood Cell Count (WBC), ×10^9^/L	9 (6.52, 12.34)	6.52 (5.25, 8.2)	<0.001
Aspartate Aminotransferase (AST), IU/L	24 (19, 32)	21 (16, 27)	<0.001
Alanine Aminotransferase (ALT), IU/L	24 (17, 34)	21 (15, 32)	<0.001
AST/ALT	1 (0.78, 1.38)	1 (0.7, 1.3)	<0.001
Total Protein (TP), g/L	68.6(63.5, 73.6)	65.8 (61.725, 70.5)	<0.001
Albumin (ALB), g/L	40.9 (37.1, 44.6)	40 (37.2, 42.8)	<0.001
Globulin (GLO), g/L	27.7 (24.7, 30.6)	25.8 (23, 28.9)	<0.001
Albumin/Globulin (A/G)	1.5 (1.3, 1.7)	1.5 (1.4, 1.8)	<0.001
Total Bilirubin (TBIL), μmol/L	15 (10.6, 20.7)	12.8 (9.5, 17.5)	<0.001
Direct Bilirubin (DDIL), μmol/L	5.6 (3.7, 8.2)	4.7 (3.3, 6.7)	<0.001
Indirect Bilirubin (IDIL), μmol/L	8.7 (5.6, 13.1)	7.8 (5.7, 10.9)	<0.001
Alkaline Phosphatase (ALP), IU/L	84 (68, 104)	79 (65, 96)	<0.001
Gamma-Glutamyl Transferase (GGT), IU/L	24 (16, 42)	25 (17, 39)	0.066
Urea, mmol/L	5.21 (4.15, 6.71)	5.27 (4.25, 6.54)	0.784
Creatinine (Cr), μmol/L	78 (62.5, 94)	83 (68, 97)	<0.001
Uric Acid (UA), μmol/L	251 (177, 327)	286 (229, 352)	<0.001
Cystatin C (CysC), mg/L	0.95 (0.82, 1.11)	1.02 (0.89, 1.18)	<0.001
Glucose, mmol/L	6.73 (5.5, 8.63)	5.48 (4.75, 7.06)	<0.001
Potassium (K^+^), mmol/L	3.78 (3.46, 4.1)	3.91 (3.67, 4.18)	<0.001
Sodium (Na^+^), mmol/L	140.1 (137.2, 142.8)	142.1 (139.6, 144.2)	<0.001
Chloride (Cl^−^), mmol/L	102.5 (99.3, 105.3)	104.5 (101.9, 106.7)	<0.001
Carbon Dioxide (CO_2_), mmol/L	23.1 (21.1, 25.1)	23.3 (21.5, 25.2)	0.001
Total Calcium (Ca), mmol/L	2.16 (2.07, 2.27)	2.2 (2.1, 2.29)	<0.001
Prothrombin Time (PT), s	11 (10.3, 11.8)	10.9 (10.3, 11.5)	<0.001
International Normalized Ratio (INR)	0.97 (0.92, 1.03)	0.97 (0.93, 1.03)	0.124
Prothrombin Activity (PTA), %	94.7 (84.7, 106.6)	94.3 (86, 104)	0.235
Activated Partial Thromboplastin Time (APTT), s	25.3 (22.1, 28.8)	25.7 (23.1, 28.8)	<0.001
Thrombin Time (TT), s	17.6 (16.3, 18.6)	18 (17, 19)	<0.001
Fibrinogen (FIB), g/L	2.92 (2.42, 3.71)	2.83 (2.36, 3.44)	<0.001
Fibrinogen Degradation Products (FDP), μg/mL	2.58 (1.52, 6.65)	1.68 (1.2, 2.8)	<0.001
D-Dimer, mg/mL	0.66 (0.28, 2.06)	0.33 (0.19, 0.75)	<0.001

ICH, intracerebral hemorrhage; IS, acute ischemic stroke.

### Selection of predictors via multiple ML algorithms

To further identify potential features capable of distinguishing stroke subtypes, we conducted multistage screening of 47 significantly differential features. In the first stage, we jointly identified 25 features among the top 30 features ranked by MI and Spearman correlation (Figure 2A). Subsequent screening yielded 13 features selected by RFECV-ADA, 15 features selected by Lasso, and 18 features selected by Boruta (Figures 2B–D; Supplementary Table 2). The optimal candidate feature subset, comprising 10 features jointly identified across methods (Figure 2E), includes CysC, K^+^, Cl^−^, Na^+^, UA, TP, WBC, NEUT%, FDP, and D-Dimer. A heatmap of the interfeature correlations revealed a strong positive correlation between D-Dimer and FDP, while the other features showed weak correlations (Figure 2F).

**Figure 2:**
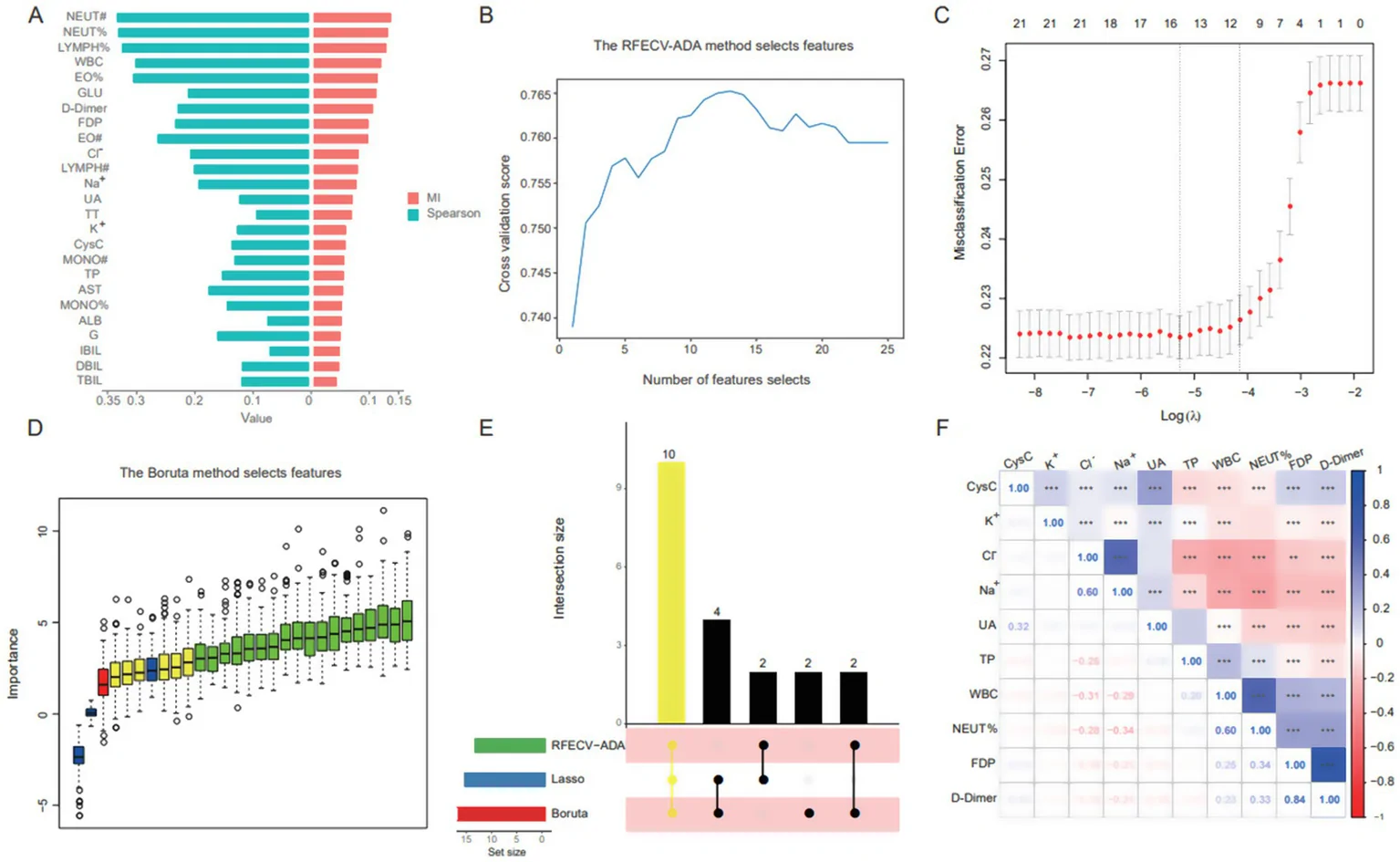
Identification of ten candidate features using a double-stage feature screening strategy. **(A)** MI and Spearman correlation were used to screen features. **(B)** RFECV-ADA was used to screen candidate features. **(C)** The Lasso algorithm was used to screen candidate features. **(D)** The Boruta algorithm was used to screen candidate features. **(E)** Venn diagram showing the intersection of the features obtained with the three algorithms (RFECV-ADA, Lasso, Boruta). **(F)** Heatmaps between candidate features. MI, mutual information; RFECV-ADA, AdaBoost-based Recursive Feature Elimination with Cross-Validation; Lasso, least absolute shrinkage and selection operator; Cl^−^, chloride; CysC, cystatin C; FDP, fibrinogen degradation product; K^+^, potassium; Na^+^, sodium; NEUT%, neutrophil percentage; TP, total protein; UA, uric acid; WBC, white blood cell count.

### Performance of various ML models for differentiating between ICH and IS

To evaluate the diagnostic performance of the candidate features for stroke subtypes, ML classification models were constructed using the aforementioned 10 candidate features. The original dataset was processed using eight resampling methods, and each resampled dataset underwent tenfold cross-validation, resulting in a total of 48 models. Model performance evaluation (Table 2; Figure 3A; Supplementary Tables 3, 4) revealed that in the test cohort, the XGBoost, GBDT, and LightGBM algorithms demonstrated consistently high performance across multiple resampling schemes, with AUC values exceeding 0.8. The model defined IS as the positive class and ICH as the negative class. Given that LightGBM exhibited the optimal overall development time, approximately 5 s (see Supplementary Table 5), it was selected as the core algorithm. Notably, under the BS2 resampling scheme, the test cohort AUC reached 0.926, and SEN and SPE were 83.82 and 87.17%, respectively. Furthermore, the corresponding BS2_LightGBM_V10 model achieved a high F1 score of 85.25% in the test cohort, and its PR (precision–recall) curve also showed excellent performance (Figures 3B,C). Additionally, the diagnostic performance of the combined 10-feature prediction (AUC = 0.926) was markedly superior to that of the single-feature prediction (Figure 3D). Consequently, the BS2_LightGBM_V10 model was identified as the final model. The version information of the software packages used in model development is summarized in Supplementary Table 6.

**Table 2:** Performance of LightGBM algorithm.

Cohort	Resampling method	SEN	SPE	ACC	PPV	NPV	F1	AUC
Training cohort	ADASYN	96.64%	94.72%	95.67%	94.73%	96.64%	95.68%	0.989 (0.987–0.991)
	BS1	96.35%	94.85%	95.60%	94.92%	96.29%	95.63%	0.989 (0.987–0.991)
	BS2	96.53%	95.10%	95.81%	95.17%	96.48%	95.84%	0.989 (0.987–0.991)
	SMOTE	96.79%	94.21%	95.50%	94.35%	96.70%	95.55%	0.989 (0.987–0.991)
	CC	99.69%	99.11%	99.40%	99.12%	99.69%	99.40%	0.995 (0.993–0.997)
	ENN	99.94%	96.89%	98.76%	98.08%	99.89%	99.00%	0.995 (0.994–0.996)
	NM	100.00%	98.53%	99.26%	98.55%	100.00%	99.27%	0.995 (0.993–0.997)
	RU	99.76%	97.81%	98.79%	97.85%	99.76%	98.8%	0.995 (0.993–0.997)
Test cohort	ADASYN	84.71%	85.43%	85.07%	85.09%	85.06%	84.91%	0.921 (0.917–0.925)
	BS1	83.59%	85.83%	84.71%	85.5%	83.95%	84.53%	0.921 (0.917–0.925)
	BS2	83.82%	87.17%	85.49%	86.72%	84.34%	85.25%	0.926 (0.922–0.93)
	SMOTE	84.49%	84.6%	84.54%	84.58%	84.5%	84.53%	0.919 (0.915–0.923)
	CC	74.77%	75.69%	75.23%	75.47%	75.00%	75.12%	0.832 (0.822–0.842)
	ENN	89.40%	72.62%	82.94%	83.91%	81.10%	86.57%	0.890 (0.883–0.897)
	NM	88.00%	80.00%	84.00%	81.48%	86.96%	84.62%	0.904 (0.896–0.912)
	RU	75.38%	72.92%	74.15%	73.57%	74.76%	74.45%	0.809 (0.798–0.82)

ACC, accuracy; AUC, area under the curve; BS1, BorderlineSMOTE-1; BS2, BorderlineSMOTE-2; CC, ClusterCentroids; ENN, EditedNearestNeighbours; LightGBM, light gradient boosting machine; NPV, negative predictive value; PPV, positive predictive value; RU, RandomUnderSampler; SEN, sensitivity; SPE, specificity.

**Figure 3:**
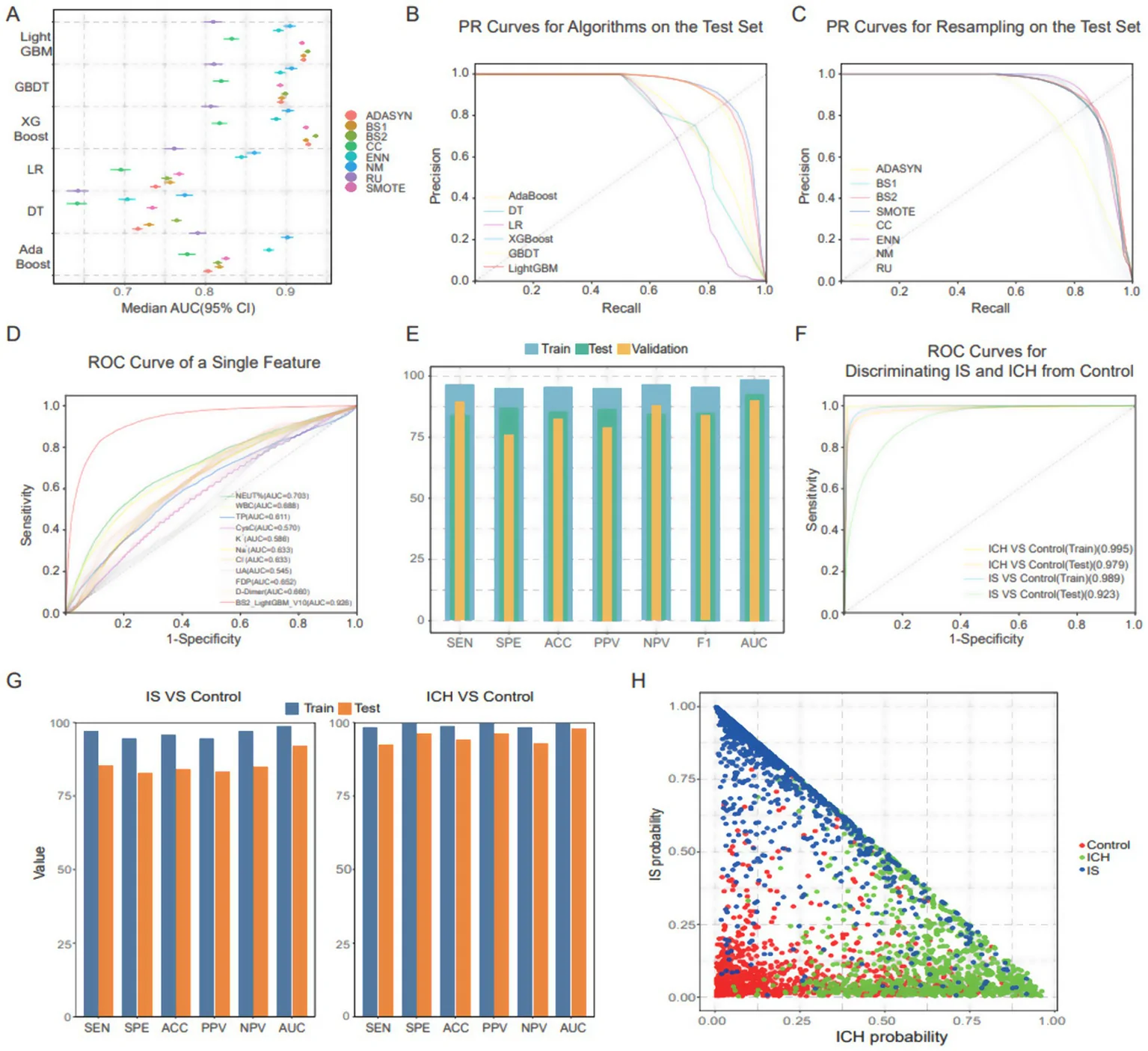
Diagnostic value of the candidate features in stroke subtypes. **(A)** AUC (95% CI) of models combining 8 resampling techniques with 6 ML algorithms on the test cohort. **(B)** PR curves of 6 ML algorithms with BS2 oversampling on the test cohort. **(C)** PR curves of 8 resampling techniques with LightGBM algorithms on the test cohort. **(D)** ROC curves of individual features and BS2_LightGBM_V10 for differentiating ICH from IS. **(E)** Performance of BS2_LightGBM_V10 in the training, test, and validation cohorts for differentiating ICH from IS. **(F)** ROC curves of BS2_LightGBM_V10 for ICH vs. control and IS vs. control classification. **(G)** Performance of BS2_LightGBM_V10 for ICH vs. control and IS vs. control classification. **(H)** Multiclass performance of BS2_LightGBM_V10: ICH vs. IS vs. control. ACC, accuracy; AdaBoost, adaptive boosting; AUC, area under the curve; BS1, BorderlineSMOTE-1; BS2, BorderlineSMOTE-2; CC, ClusterCentroids; Cl^−^, chloride; CysC, cystatin C; DT, decision tree; ENN, EditedNearestNeighbours; FDP, fibrinogen degradation product; ICH, Intracerebral hemorrhage; IS, ischemic stroke; IVW, inverse variance weighting; K^+^, potassium; LightGBM, light gradient boosting machine; LR, logistic regression; Na^+^, sodium; NEUT%, neutrophil percentage; NM, NearMiss; NPV, negative predictive value; PR, precision-recall; PPV, positive predictive value; SEN, sensitivity; SPE, specificity; TP, total protein; UA, uric acid; WBC, white blood cell count; XGBoost, eXtreesme Gradient Boosting.

### Validation of model performance with a temporal validation cohort

Furthermore, an independent cohort comprising 153 ICH patients and 342 IS patients was used for temporal validation (Supplementary Table 7). The BS2_LightGBM_V10 model also demonstrated excellent performance in the temporal validation cohort, with an AUC of 0.902, ACC of 82.8%, and an F1 score of 83.9% for distinguishing between IS and ICH, with SEN of 89.5%, SPE of 76.1%, a PPV of 78.9%, and an NPV of 87.9% (Figure 3E). The model results on the non-resampled temporal validation cohort also showed good performance (Supplementary Table 8). Calibration evaluation of the model on the non-resampled temporal cohort yielded a Brier score of 0.1354, which is below the benchmark threshold of 0.25, indicating that the model’s predicted probabilities have good overall accuracy. The calibration slope was 0.88 (ideal value = 1), and the calibration intercept was 0.20 (ideal value = 0), with the calibration curve lying essentially along the 45° diagonal line (Supplementary Figure 1). These results demonstrate good agreement between the model’s predicted probabilities and the observed frequencies, suggesting that the calibration performance is within a clinically acceptable range. These results indicate that the BS2_LightGBM_V10 diagnostic model provides a promising preliminary tool for differentiating between IS and ICH.

### Performance of the BS2_LightGBM_V10 model in differentiating among ICH patients, IS patients and controls

Additionally, the performance of the BS2_LightGBM_V10 model in distinguishing stroke subtypes from controls was evaluated in this study. The model demonstrated high performance in both tasks (ICH vs. controls and IS vs. controls), achieving AUC values exceeding 0.9 in both the training and test cohorts (Figure 3F). Specifically, for distinguishing ICH patients from controls, SEN, SPE, ACC, PPV, and NPV all exceeded 92% in the test cohort. The performance metrics for differentiating IS patients from controls presented relatively low values but remained robust, with SEN, SPE, ACC, PPV, and NPV all above 82% in the test cohort (Figure 3G; Supplementary Table 9). Notably, in the three-class classification task (distinguishing among ICH, IS, and controls), the model attained a test cohort accuracy of 79.07% (Figure 3H). Collectively, these results demonstrate that the final model exhibits favorable discriminative ability in both binary classification tasks (ICH vs. controls, IS vs. controls) and the three-class classification task (ICH, IS, controls).

### SHAP-based visualization of feature importance

To elucidate the contribution of individual variables within the model, we employed SHAP visualization for both global and local explanations. According to the density plot (Figure 4A), individuals with lower NEUT% values exhibited a higher probability of being diagnosed with IS than those with higher values did. Conversely, individuals with higher K^+^ values were more likely to have an IS diagnosis than those with lower values were. Furthermore, we evaluated the specific contributions of the selected features to the prediction. The five most influential features were NEUT%, CysC, D-Dimer, TP, and Na^+^ (Figure 4B). SHA*p* values reveal the contribution of each feature to the final prediction, effectively clarifying and explaining the model’s predictions for individual patients. Moreover, a representative case study is provided to demonstrate the interpretability of the model (Figure 4C).

**Figure 4:**
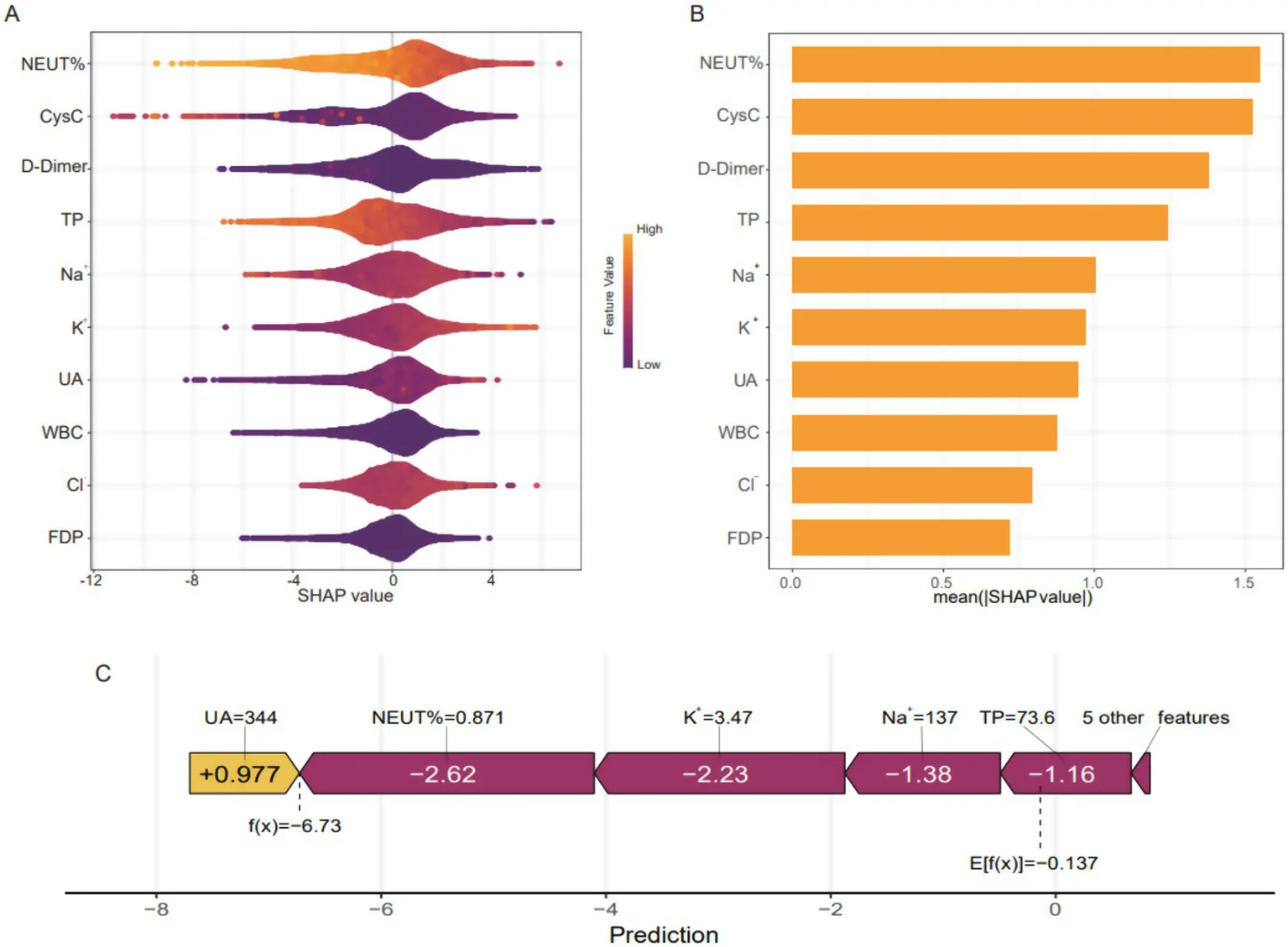
A SHAP diagram was used for model interpretation. SHAP values in the BS2_LightGBM_V10 model. **(A)** SHAP summary plot showing the effects of features on model output. **(B)** SHAP bar plot illustrating the mean SHAP values for each feature. **(C)** SHAP force plot (individual-level interpretation). Cl^−^, chloride; CysC, cystatin C; FDP, fibrinogen degradation product; K^+^, potassium; Na^+^, sodium; NEUT%, neutrophil percentage; TP, total protein; UA, uric acid; WBC, white blood cell count.

### Implementation of the graphical user interface

In this study, an interactive visualization system for the predictive model based on the PyQt5 framework was developed (Figure 5). Its core functional modules include a data input and preprocessing interface (Figure 5A) and a real-time risk assessment and probability visualization interface (Figure 5B). The implementation code is publicly available at https://github.com/haoxiaoyan/STROKE.

**Figure 5:**
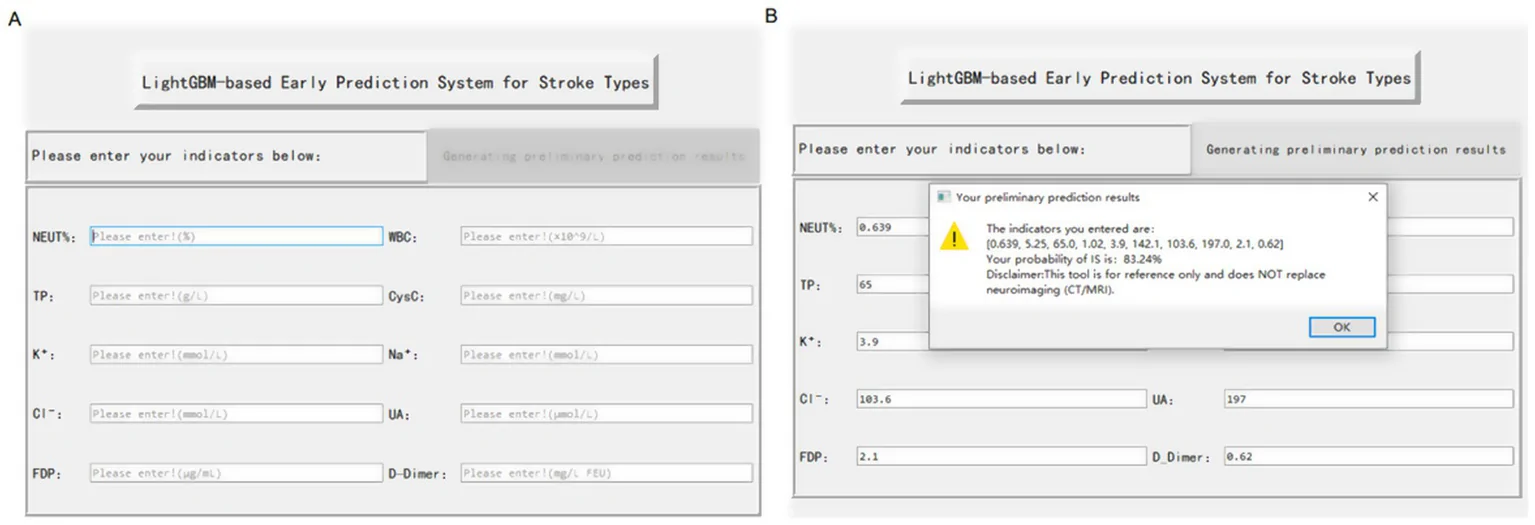
Clinical prediction interface developed with PyQt5. **(A)** Data input interface. **(B)** Real-time prediction interface. Cl^−^, chloride; CysC, cystatin C; FDP, fibrinogen degradation product; K^+^, potassium; Na^+^, sodium; NEUT%, neutrophil percentage; TP, total protein; UA, uric acid; WBC, white blood cell count; LightGBM, light gradient boosting machine; IS, ischemic stroke.

### Causal effects of the nine laboratory indicators on ICH and IS

Furthermore, we investigated the causal relationships between the nine indicators and patients with ICH and IS. LDSC was employed to estimate the genetic correlations between the exposure factors and stroke outcomes (Supplementary Table 10). In this study, genetic variants strongly associated with blood phenotypic traits were selected as IVs for the exposure factors. Specifically, 309, 15, 8, 185, 267, 251, 500, 354, and 12 IVs were selected for CysC, K^+^, Cl^−^, Na^+^, UA, TP, WBC, NEUT%, and D-Dimer, respectively (Supplementary Table 11). IVW analysis revealed that UA had potential causal associations with both ICH and IS (*p* < 0.05), whereas CysC and K^+^ showed potential causal associations only with IS. These positive findings demonstrated that elevated UA levels were associated with an increased risk of ICH (OR = 1.309, *p* < 0.001), and that elevated levels of UA (OR = 1.0008, *p* = 0.027), CysC (OR = 1.0011, *p* = 0.015), and K^+^ (OR = 1.004, *p* = 0.032) were associated with an increased risk of IS (Figure 6; Supplementary Table 12). Collectively, these findings suggest potential genetic correlations between UA, CysC, K^+^ and IS, as well as between UA and ICH.

**Figure 6:**
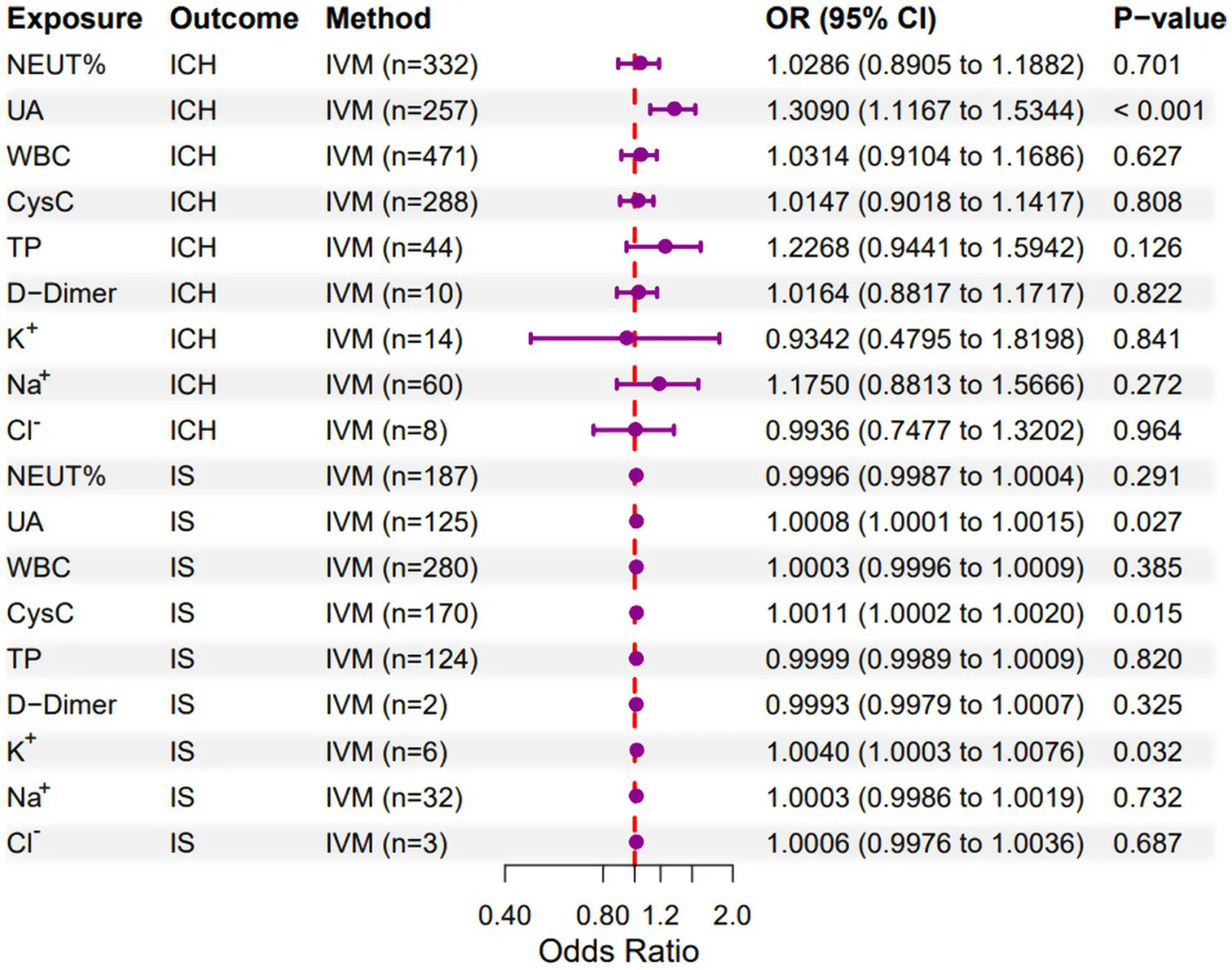
Forest plot for the causal effects of blood biomarkers on ICH or IS derived from IVW. ACC accuracy. Cl^−^, chloride; CysC, cystatin C; FDP, fibrinogen degradation product; K^+^, potassium; Na^+^, sodium; NEUT%, neutrophil percentage; TP, total protein; UA, uric acid; WBC, white blood cell count; IS, ischemic stroke; ICH, Intracerebral hemorrhage; IVW, inverse variance weighting.

### Sensitivity analysis

We conducted sensitivity analyses to assess the robustness of the significant causal associations (Supplementary Table 13). Cochran’s *Q* test results from both the IVW and MR-Egger methods indicated no significant heterogeneity (*p* > 0.05). Furthermore, the MR-Egger regression intercepts did not reveal significant directional pleiotropy (*p* > 0.05). The MR-PRESSO test, which uses 1,000 simulations, also revealed no significant horizontal pleiotropy (*p* > 0.05). Additionally, leave-one-out analysis confirmed that no single SNP drove the causal estimates (Supplementary Figures 2–5). Collectively, these sensitivity analyses demonstrate the reliability and robustness of our causal inferences.

### Colocalization analyses

We conducted a colocalisation analysis to identify genes associated with the positive exposure factors (UA, CysC and K^+^) and ICH or IS. The colocalisation analysis revealed that serum UA shared causal genetic variants with ICH at a specific locus on chromosome 12 (PP.H4 > 0.8) (Supplementary Table 14). Eighteen protein-coding genes were annotated within this locus, including CCDC63, ALDH2, PTPN11, MYL2, MAPKAPK5, RPH3A, CUX2, TMEM116, PHETA1, ERP29, SH2B3, NAA25, ATXN2, TRAFD1, BRAP, HECTD4, ACAD10, and RPL6 (Figure 7). However, fine mapping and functional studies are still required to pinpoint the true causal gene(s) at this locus.

**Figure 7:**
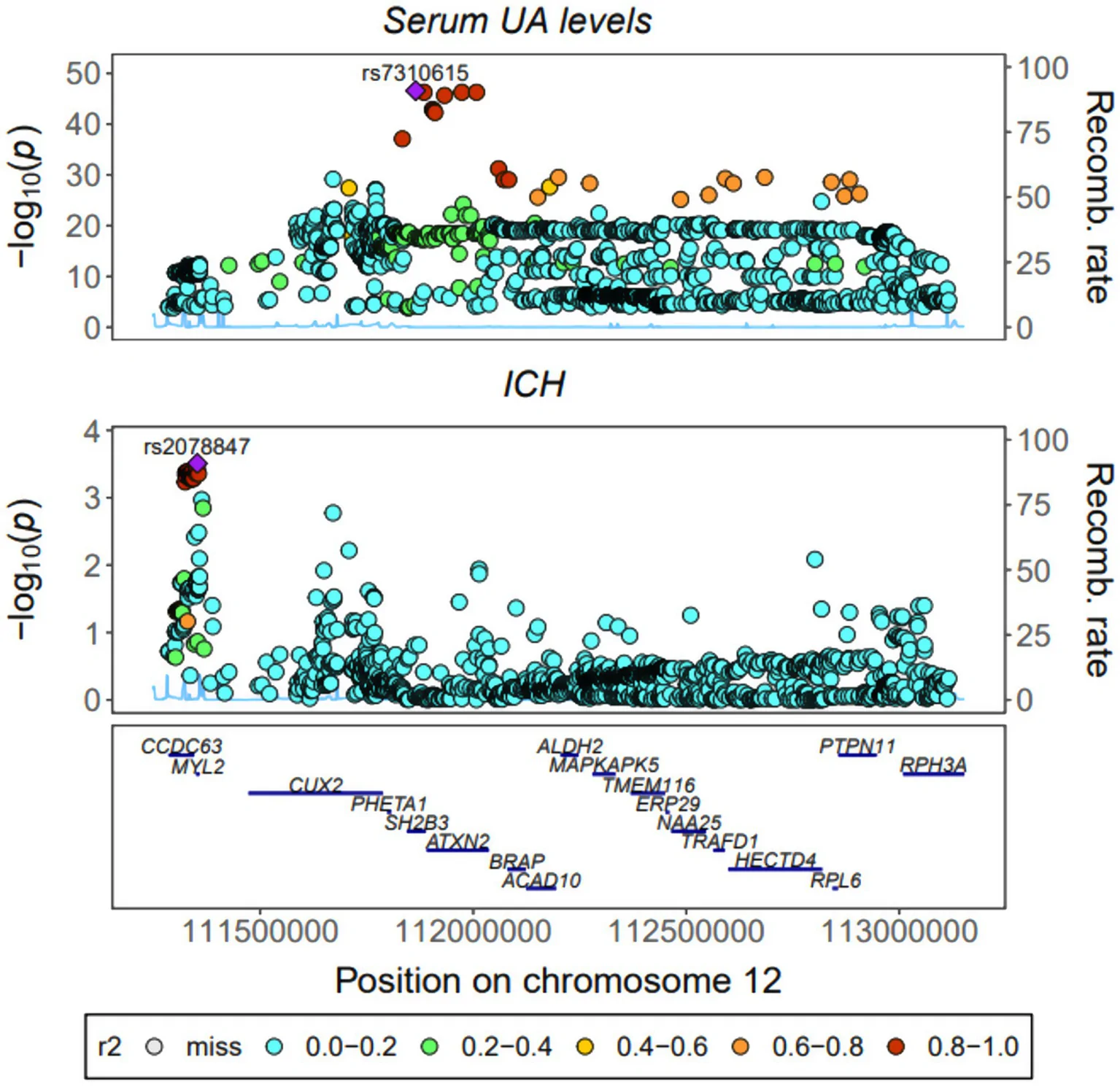
Colocalization analysis results for UA levels and ICH. LocusZoom plots illustrating evidence of genetic colocalization revealed 18 genes associated with UA levels and ICH. ICH, Intracerebral hemorrhage; UA, uric acid.

## Discussion

Given the high incidence and mortality rates of stroke globally, coupled with significant differences in treatment strategies for distinct stroke subtypes and the current lack of widely applicable biomarkers in clinical practice, there is urgent need to identify potential markers for distinguishing between ICH and IS.

In this study, we employed a retrospective cohort design to propose and validate, a novel ML-based predictive model for the differential diagnosis of stroke subtypes. Through a systematic analysis of laboratory data from patients with ICH and IS, combined with a two-stage data-driven strategy, we identified ten key laboratory features derived from differentially expressed variables. To determine the optimal algorithmic combination, we comprehensively evaluated the performance of multiple resampling techniques paired with ML algorithms and rigorously evaluated their efficacy via cross-validation. Finally, after comprehensively considering model discriminative performance and development efficiency, we identified the optimal combination with BS2 as the resampling strategy and LightGBM as the core classification algorithm, which achieves a balance between predictive accuracy and development efficiency. The integrated BS2_LightGBM_V10 model demonstrated good discriminative power: for distinguishing ICH patients from IS patients, ICH patients from controls, and IS patients from controls, the AUC values were 0.902, 0.979, and 0.923, respectively. The corresponding SEN values were 89.47%, 92.52%, and 85.6%, while the SPE values were 76.11%, 96.21%, and 82.81%. This model exhibits favorable discriminatory performance and real-time predictive ability.

During the feature engineering phase, MI and Spearman correlation analysis were employed for the initial screening of differential variables. To optimize the feature subset and enhance the discriminative power and generalizability of the model, three refined feature selection algorithms—RFECV-AdaBoost, Boruta, and Lasso—were rigorously applied. Following this stringent screening pipeline, we ultimately identified and retained ten core laboratory features with high discriminative value: WBC, NEUT%, CysC, UA, TP, FDP, D-Dimer, K^+^, Cl^−^, and Na^+^. To determine the potential biological significance of these features in distinguishing ICH from IS, we conducted an in-depth analysis. WBC and NEUT% were significantly greater in ICH patients than in IS patients, indicating a more pronounced acute inflammatory response following ICH (37). In contrast, the inflammatory response in IS was usually less severe, although patients with secondary infections or large-area infarction were excluded. CysC tended to increase in IS patients, which was correlated with their increased incidence of chronic kidney injury risk factors (38, 39). As an acute event, ICH typically causes only mild renal impairment unless the patient has preexisting comorbidities. UA, closely associated with atherosclerosis, oxidative stress, and inflammatory cascades, constituted a risk factor for IS but demonstrated weaker pathological relevance to ICH (40, 41). TP, an indicator of hepatic synthetic function, will require further investigation to elucidate its precise mechanism in differential diagnosis. FDP and D-Dimer levels were significantly greater in the ICH group than in the IS group, which may be attributed to excessive activation of the coagulation system and secondary hyperfibrinolysis triggered by vascular rupture. Electrolyte analysis revealed that ICH was more frequently complicated by two specific disorders, namely, hyponatremia (42), which is primarily due to hypothalamic or pituitary damage causing SIADH or cerebral salt-wasting syndrome, and hypokalemia (43), which is mainly attributed to stress-induced sympathetic activation, catecholamine surge, and glucocorticoid secretion. These factors activate β2-receptors linked to Na^+^-K^+^-ATPase activity, promoting K^+^ influx into cells and, consequently, serum K^+^ depletion. Cl^−^ levels covaried with Na^+^ levels. Notably, while individual biomarkers have previously been explored, no prior studies have systematically integrated and validated the diagnostic efficacy of this multifeature combined model for distinguishing ICH from IS.

With the emergence of ML as a novel research paradigm in medical diagnostic modeling, ML algorithms have become effective tools for mining high-dimensional laboratory data. We employed a systematic comparison strategy to evaluate the diagnostic efficacy of the aforementioned ten biomarkers by combining eight resampling techniques and six ML classification algorithms. LightGBM, widely utilized in prior research, is particularly well suited for processing large-scale datasets. As a highly efficient variant of the GBDT framework, its core mechanism involves iteratively constructing weak learners (shallow decision trees) to fit the residual gradient direction and ultimately integrating them into a weighted predictive model. Building upon the core GBDT framework, this algorithm implements several computational optimizations: GOSS (gradient-based one-sided sampling) reduces the data volume, EFB (exclusive feature bundling) decreases feature dimensionality, and a histogram-based algorithm accelerates feature split point calculation. These innovations enable the algorithm to significantly outperform conventional GBDT implementations (e.g., XGBoost) in terms of computational efficiency and memory consumption while maintaining or even enhancing predictive accuracy.

In the field of rapid assessment for acute cerebrovascular diagnosis and treatment, various decision support tools based on different data modalities have been explored. For example, recent studies have utilized readily available physiological parameters and electrocardiographic indicators for prognostic assessment of acute ischemic stroke (44), while clinical risk scores have also demonstrated their value in predicting short-term events after transient ischemic attack (45). Compared with the above approaches, the unimodal routine laboratory indicators adopted in this study have advantages such as high objectivity, low testing costs, and rapid accessibility in most medical institutions. However, different data modalities (physiological parameters, electrocardiographic indicators, and laboratory indices) may offer complementary value, and multimodal fusion strategies could be explored in the future to further enhance the comprehensiveness of early decision support.

The MR analysis conducted in this study revealed potential causal associations between four blood biomarkers and stroke subtypes. However, subsequent colocalization analysis supported only the association between UA and ICH. With respect to the remaining associations (of UA, CysC, and K^+^ with IS) where MR signals were detected but colocalization evidence was lacking, the discrepancy may be attributed to methodological and biological factors. One explanation is that the genetic instruments point to distinct causal variants. While MR estimates the causal effect on the basis of the overall association of valid IVs (SNPs) with both exposure and outcome, colocalization specifically tests whether they share the same causal variant within a genomic region. Since the MR results in this study are based on GWAS summary statistics of European ancestry, the conclusions may not be directly extrapolated to East Asian populations, owing to potential population differences in allele frequencies, linkage disequilibrium patterns, and effect sizes.

The core contributions of this study are the development and validation of an ML-based stroke subtype discrimination model (BS2_LightGBM_V10). The results from a temporal validation cohort confirmed favorable predictive performance of the model in distinguishing ICH from IS, with an AUC of 0.902. To advance clinical translation, we developed a visualization platform using the PyQt5 framework, featuring a user-friendly graphical interface. The role of this model is to provide laboratory data-driven decision support, rather than to replace neuroimaging itself as an independent diagnostic confirmation tool.

It should be noted that the control group in this study consisted solely of patients with uncomplicated hypertension. Consequently, the reported AUC values for ICH versus controls and IS versus controls reflect the model’s ability to differentiate “stroke” from “stable hypertension” specifically within a hypertensive population, and cannot be extrapolated to the general population or to emergency differential diagnostic settings. This design does not permit the conclusion that the identified indicators would perform equally well in non-hypertensive or normotensive individuals. Future studies incorporating more diverse control groups, including normotensive subjects and patients with other acute conditions, are warranted to further validate the generalisability and specificity of the model. In addition, the MR analysis in this study has certain limitations. The exposure GWAS for CysC, TP, and K^+^, and the outcome GWAS for IS, all include UK Biobank samples, raising the potential for sample overlap. Although the genetic instruments used in this study all have sufficient statistical power (*F*-statistic > 10), the potential bias introduced by sample overlap cannot be completely ruled out. Therefore, we have maintained a cautious interpretation of our findings.

Notably, this study has several limitations. The current temporal validation falls short of genuine external validation, for which multi-center and multi-regional studies remain essential; the case–case design and the absence of stroke mimics confine the model’s applicability to differentiating ICH from IS among hospitalized stroke patients, leaving its performance in the full spectrum of emergency presentations unevaluated; moreover, our cohort lacks complete data on stroke severity assessment, TOAST etiologic classification, and lesion parameters, and therefore the performance of the model in patients with embolic infarction, large-vessel disease, small-vessel disease, hypertensive deep hemorrhage, lobar hemorrhage, or coma remains unclear, warranting further validation in prospective studies; it remains uncertain whether the first blood sample was collected prior to diagnostic imaging and treatment (including anticoagulation reversal and reperfusion therapy); the uncertain time from admission to the first sampling may compromise the stability of time-dependent biomarkers, constituting an uncontrolled confounder; the extended development period raises concerns about potential shifts in laboratory platforms and clinical practices, which could introduce systematic bias—although we have preliminarily explored temporal stability, its impact has not been fully quantified; and the missing data mechanism was not formally tested for randomness. We plan to address these limitations in subsequent investigations.

## Conclusion

In conclusion, in this study, we pioneered the integration of ten routine laboratory biomarkers to construct the BS2_LightGBM_V10 prediction model. When validated in a temporal validation cohort, the model demonstrated high discriminative efficacy for distinguishing ICH and IS, serving as a tool to assist subtype differentiation.

## Data Availability

The original contributions presented in the study are included in the article/Supplementary material, further inquiries can be directed to the corresponding author.

## Footnotes

1
https://gwas.mrcieu.ac.uk/datasets/ebi-a-GCST90018870/

2
https://gwas.mrcieu.ac.uk/datasets/ukb-b-19350/

3
https://github.com/chr1swallace/coloc/

## Glossary

ACC: accuracy
AdaBoost: adaptive boosting
AUC: area under the curve
BS1: BorderlineSMOTE-1
BS2: BorderlineSMOTE-2
CC: ClusterCentroids
Cl: chloride
CysC: cystatin C
DT: decision tree
ENN: EditedNearestNeighbours
FDP: fibrinogen degradation product
ICH: Intracerebral hemorrhage
IS: ischemic stroke
IVW: inverse variance weighting
K^+^: potassium
Lasso: least absolute shrinkage and selection operator
LightGBM: light gradient boosting machine
LR: logistic regression
MI: mutual information
ML: machine learning
MR-PRESSO: MR pleiotropy residual sum
MR: Mendelian randomization
Na^+^: sodium
NEUT%: neutrophil percentage
NM: NearMiss
NPV: negative predictive value
PR: precision-recall
PPV: positive predictive value
RFECV-ADA: AdaBoost-based Recursive Feature Elimination with Cross-Validation
RU: RandomUnderSampler
SEN: sensitivity
SHAP: SHapley Additive exPlanations
SPE: specificity
SMOTE: synthetic minority oversampling technique
TP: total protein
UA: uric acid
WBC: white blood cell count
XGBoost: eXtreesme Gradient Boosting
