# Supplementary material for: Routine laboratory biomarker signature combined with machine learning for rapid differentiation of intracerebral hemorrhage from acute ischemic stroke
Source: Front Neurol. 2026 Oct 15;17:1931764. doi: 10.3389/fneur.2026.1931764 (PMC13716555; doi:10.3389/fneur.2026.1931764)
Supplement: Supplementary file 2 [file Data_Sheet_2.ZIP › Supplemental Table 1-3.docx]

**Supplementary Table 1. Summary Table of GWAS dataset for blood indicators**

| Phenotypes | Sample size | SNP | Download file name |
| --- | --- | --- | --- |
| WBC | 563,946 | NA | https://gwas.mrcieu.ac.uk/datasets/ieu-b-30/ |
| NEUT% | 408,112 | 40,312,502 | https://gwas.mrcieu.ac.uk/datasets/ebi-a-GCST90002399/ |
| CysC | 344,264 | 13,586,047 | https://gwas.mrcieu.ac.uk/datasets/ukb-d-30720_irnt/ |
| UA | 343,836 | 190,412,86 | https://gwas.mrcieu.ac.uk/datasets/ebi-a-GCST90018977/ |
| TP | 314,921 | 13,585,298 | https://gwas.mrcieu.ac.uk/datasets/ukb-d-30860_irnt/ |
| K^+^ | 64,979 | 9,851,867 | https://gwas.mrcieu.ac.uk/datasets/ukb-b-17881/ |
| Na^+^ | 421,132 | NA | https://www.ebi.ac.uk/gwas/studies/GCST90479475 |
| Cl^-^ | 1,206 | NA | https://www.ebi.ac.uk/gwas/studies/GCST009170 |
| D-Dimer | 3,301 | 10,534,735 | https://gwas.mrcieu.ac.uk/datasets/prot-a-1086/ |

**Supplementary Table 2. Indicators selected by multiple methods**

| RFECV-ADA | Lasso | Boruta |
| --- | --- | --- |
| NEUT% | NEUT% | NEUT% |
| LYMPH# | EO% | LYMPH% |
| WBC | K^+^ | EO% |
| TP | LYMPH% | NEUT# |
| CysC | NEUT# | LYMPH# |
| K^+^ | WBC | EO# |
| Na^+^ | TP | WBC |
| Cl^-^ | Cl^-^ | ALB |
| UA | D-Dimer | TP |
| TT | G | G |
| FDP | Na^+^ | CysC |
| D-Dimer | CysC | K^+^ |
| GLU | TT | Na^+^ |
|  | UA | Cl^-^ |
|  | FDP | UA |
|  |  | FDP |
|  |  | D-Dimer |
|  |  | GLU |

**Supplementary Table 3.** **Performance of five ML models**

|  |  | **Training cohort** | | | | | | | **Test cohort** | | | | | | |
| --- | --- | --- | --- | --- | --- | --- | --- | --- | --- | --- | --- | --- | --- | --- | --- |
|  |  | **SEN** | **SPE** | **ACC** | **PPV** | **NPV** | **F1** | **AUC** | **SEN** | **SPE** | **ACC** | **PPV** | **NPV** | **F1** | **AUC** |
| AdaBoost | ADASYN | 73.37% | 75.62% | 74.50% | 74.70% | 74.31% | 74.03% | 0.825(0.819-0.831) | 71.99% | 73.71% | 72.86% | 72.88% | 72.84% | 72.43% | 0.803(0.797-0.809) |
|  | BS1 | 75.21% | 76.59% | 75.90% | 76.26% | 75.54% | 75.73% | 0.837(0.831-0.843) | 74.11% | 74.78% | 74.44% | 74.61% | 74.28% | 74.36% | 0.817(0.811-0.823) |
|  | BS2 | 75.65% | 75.54% | 75.60% | 75.57% | 75.62% | 75.61% | 0.836(0.83-0.842) | 74.11% | 73.99% | 74.05% | 74.02% | 74.08% | 74.06% | 0.815(0.809-0.821) |
|  | SMOTE | 76.83% | 75.96% | 76.39% | 76.16% | 76.63% | 76.49% | 0.845(0.839-0.851) | 75.45% | 74.33% | 74.89% | 74.61% | 75.17% | 75.03% | 0.825(0.819-0.831) |
|  | CC | 76.45% | 72.80% | 74.62% | 73.75% | 75.56% | 75.07% | 0.831(0.821-0.841) | 72.00% | 68.31% | 70.15% | 69.44% | 70.93% | 70.70% | 0.769(0.758-0.78) |
|  | ENN | 91.66% | 73.10% | 84.51% | 84.47% | 84.60% | 87.92% | 0.910(0.904-0.916) | 89.79% | 70.15% | 82.23% | 82.77% | 81.14% | 86.15% | 0.878(0.871-0.885) |
|  | NM | 92.17% | 80.49% | 86.33% | 82.53% | 91.14% | 87.08% | 0.935(0.929-0.941) | 88.92% | 78.15% | 83.54% | 80.28% | 87.59% | 84.38% | 0.901(0.893-0.909) |
|  | RU | 79.8% | 73.04% | 76.42% | 74.74% | 78.33% | 77.18% | 0.847(0.837-0.857) | 76.0% | 68.92% | 72.46% | 70.98% | 74.17% | 73.39% | 0.790(0.779-0.801) |
| DT | ADASYN | 94.34% | 96.91% | 95.63% | 96.77% | 94.57% | 95.54% | 0.988(0.986-0.99) | 68.3% | 75.25% | 71.81% | 73.03% | 70.75% | 70.59% | 0.716(0.709-0.723) |
|  | BS1 | 94.61% | 97.40% | 96.00% | 97.33% | 94.76% | 95.95% | 0.989(0.987-0.991) | 71.09% | 76.12% | 73.61% | 74.85% | 72.48% | 72.92% | 0.730(0.723-0.737) |
|  | BS2 | 94.46% | 97.06% | 95.76% | 96.98% | 94.6% | 95.7% | 0.989(0.987-0.991) | 72.66% | 80.02% | 76.34% | 78.43% | 74.53% | 75.43% | 0.764(0.757-0.771) |
|  | SMOTE | 95.85% | 97.01% | 96.43% | 96.98% | 95.90% | 96.41% | 0.991(0.99-0.992) | 71.2% | 76.00% | 73.6% | 74.8% | 72.52% | 72.95% | 0.734(0.727-0.741) |
|  | CC | 97.65% | 95.76% | 96.71% | 95.84% | 97.60% | 96.74% | 0.992(0.99-0.994) | 66.46% | 64.62% | 65.54% | 65.26% | 65.83% | 65.85% | 0.641(0.628-0.654) |
|  | ENN | 99.55% | 93.20% | 97.10% | 95.90% | 99.24% | 97.69% | 0.988(0.986-0.99) | 82.43% | 65.23% | 75.80% | 79.07% | 69.97% | 80.72% | 0.703(0.692-0.714) |
|  | NM | 99.00% | 89.47% | 94.24% | 90.39% | 98.90% | 94.49% | 0.968(0.964-0.972) | 85.54% | 73.85% | 79.69% | 76.58% | 83.62% | 80.78% | 0.774(0.763-0.785) |
|  | RU | 98.46% | 95.45% | 96.96% | 95.59% | 98.41% | 97.00% | 0.991(0.989-0.993) | 66.77% | 64.62% | 65.69% | 65.36% | 66.04% | 66.05% | 0.642(0.629-0.655) |
| LR | ADASYN | 70.78% | 64.67% | 67.70% | 66.29% | 69.28% | 68.46% | 0.739(0.732-0.746) | 70.76% | 64.73% | 67.72% | 66.32% | 69.28% | 68.46% | 0.738(0.731-0.745) |
|  | BS1 | 72.25% | 66.22% | 69.24% | 68.14% | 70.47% | 70.14% | 0.756(0.749-0.763) | 72.21% | 66.29% | 69.25% | 68.18% | 70.46% | 70.14% | 0.756(0.749-0.763) |
|  | BS2 | 72.21% | 65.55% | 68.88% | 67.70% | 70.23% | 69.88% | 0.752(0.745-0.759) | 72.21% | 65.51% | 68.86% | 67.68% | 70.22% | 69.87% | 0.752(0.745-0.759) |
|  | SMOTE | 73.91% | 66.41% | 70.16% | 68.75% | 71.79% | 71.23% | 0.767(0.76-0.774) | 73.88% | 66.41% | 70.15% | 68.74% | 71.77% | 71.22% | 0.767(0.76-0.774) |
|  | CC | 67.15% | 62.30% | 64.73% | 64.05% | 65.48% | 65.56% | 0.697(0.684-0.71) | 66.77% | 62.15% | 64.46% | 63.82% | 65.16% | 65.26% | 0.695(0.682-0.708) |
|  | ENN | 90.19% | 62.78% | 79.63% | 79.45% | 80.04% | 84.48% | 0.846(0.838-0.854) | 89.96% | 62.77% | 79.48% | 79.39% | 79.69% | 84.33% | 0.844(0.836-0.852) |
|  | NM | 85.47% | 72.97% | 79.22% | 75.97% | 83.4% | 80.43% | 0.861(0.852-0.87) | 85.54% | 72.92% | 79.23% | 75.96% | 83.45% | 80.45% | 0.860(0.851-0.869) |
|  | RU | 74.71% | 65.48% | 70.10% | 68.4% | 72.14% | 71.41% | 0.763(0.751-0.775) | 74.46% | 65.54% | 70.00% | 68.36% | 71.96% | 71.26% | 0.761(0.749-0.773) |
| XGBoost | ADASYN | 99.45% | 98.94% | 99.2% | 98.93% | 99.46% | 99.19% | 0.995(0.994-0.996) | 84.15% | 86.31% | 85.24% | 85.78% | 84.73% | 84.96% | 0.927(0.923-0.931) |
|  | BS1 | 98.66% | 97.91% | 98.29% | 97.92% | 98.65% | 98.29% | 0.994(0.993-0.995) | 83.26% | 86.16% | 84.71% | 85.75% | 83.73% | 84.48% | 0.922(0.918-0.926) |
|  | BS2 | 99.60% | 99.10% | 99.35% | 99.1% | 99.6% | 99.35% | 0.995(0.994-0.996) | 84.15% | 88.95% | 86.55% | 88.39% | 84.88% | 86.22% | 0.935(0.931-0.939) |
|  | SMOTE | 99.21% | 98.45% | 98.83% | 98.46% | 99.20% | 98.83% | 0.995(0.994-0.996) | 84.26% | 85.49% | 84.88% | 85.31% | 84.45% | 84.78% | 0.923(0.919-0.927) |
|  | CC | 88.55% | 88.55% | 88.55% | 88.55% | 88.55% | 88.55% | 0.954(0.949-0.959) | 73.23% | 74.46% | 73.85% | 74.14% | 73.56% | 73.68% | 0.818(0.808-0.828) |
|  | ENN | 95.36% | 83.25% | 90.69% | 90.08% | 91.82% | 92.64% | 0.964(0.96-0.968) | 89.38% | 72.00% | 82.68% | 83.57% | 80.97% | 86.39% | 0.889(0.882-0.896) |
|  | NM | 94.46% | 86.06% | 90.26% | 87.14% | 93.96% | 90.64% | 0.963(0.958-0.968) | 88.31% | 79.69% | 84.00% | 81.30% | 87.21% | 84.65% | 0.909(0.902-0.916) |
|  | RU | 86.81% | 83.80% | 85.30% | 84.27% | 86.40% | 85.52% | 0.929(0.922-0.936) | 74.46% | 73.23% | 73.85% | 73.56% | 74.14% | 74.01% | 0.814(0.804-0.824) |
| GBDT | ADASYN | 94.55% | 91.38% | 92.95% | 91.50% | 94.47% | 93.00% | 0.977(0.975-0.979) | 85.6% | 83.13% | 84.36% | 83.28% | 85.47% | 84.42% | 0.915(0.911-0.919) |
|  | BS1 | 94.53% | 91.46% | 92.99% | 91.71% | 94.36% | 93.10% | 0.976(0.974-0.978) | 84.93% | 83.48% | 84.21% | 83.72% | 84.71% | 84.32% | 0.894(0.889-0.899) |
|  | BS2 | 94.80% | 91.59% | 93.20% | 91.85% | 94.63% | 93.30% | 0.977(0.975-0.979) | 85.6% | 83.71% | 84.65% | 84.01% | 85.32% | 84.8% | 0.918(0.914-0.922) |
|  | SMOTE | 94.75% | 91.17% | 92.96% | 91.48% | 94.56% | 93.09% | 0.976(0.974-0.978) | 85.27% | 82.48% | 83.87% | 82.95% | 84.84% | 84.09% | 0.911(0.907-0.915) |
|  | CC | 97.58% | 96.79% | 97.18% | 96.81% | 97.55% | 97.19% | 0.992(0.99-0.994) | 73.85% | 74.15% | 74.00% | 74.07% | 73.93% | 73.96% | 0.816(0.806-0.826) |
|  | ENN | 99.12% | 93.37% | 96.90% | 95.98% | 98.52% | 97.52% | 0.992(0.99-0.994) | 87.64% | 72.92% | 81.97% | 83.76% | 78.74% | 85.65% | 0.877(0.87-0.884) |
|  | NM | 99.79% | 97.95% | 98.87% | 97.99% | 99.79% | 98.88% | 0.995(0.993-0.997) | 85.85% | 79.69% | 82.77% | 80.87% | 84.92% | 83.29% | 0.899(0.891-0.907) |
|  | RU | 89.82% | 85.06% | 87.44% | 85.74% | 89.3% | 87.74% | 0.949(0.943-0.955) | 74.77% | 72.92% | 73.85% | 73.41% | 74.29% | 74.08% | 0.810(0.799-0.821) |
