# Supplementary material for: Routine laboratory biomarker signature combined with machine learning for rapid differentiation of intracerebral hemorrhage from acute ischemic stroke
Source: Front Neurol. 2026 Oct 15;17:1931764. doi: 10.3389/fneur.2026.1931764 (PMC13716555; doi:10.3389/fneur.2026.1931764)
Supplement: Supplementary file 2 [file Data_Sheet_2.ZIP › Supplemental Table 5.docx]

**Supplementary Table 5.** **Overall algorithm development time**

|  | **AdaBoost** | **DT** | **LR** | **XGBoost** | **GBDT** | **LightGBM** |
| --- | --- | --- | --- | --- | --- | --- |
| ADASYN | 87.17S | 6.98S | 3.66S | 24.91S | 210.67S | 5.01S |
| BS1 | 85.52S | 5.38S | 3.67S | 23.59S | 207.36S | 5.25S |
| BS2 | 85.13S | 5.87S | 3.40S | 24.24S | 205.18S | 4.86S |
| SMOTE | 85.06S | 5.31S | 3.06S | 24.68S | 204.26S | 5.31S |
| CC | 30.87S | 2.70S | 2.18S | 10.33S | 66.33S | 3.89S |
| ENN | 27.69S | 2.90S | 2.14S | 10.49S | 53.71S | 4.24S |
| NM | 39.31s | 2.46S | 2.40S | 8.1S | 44.55S | 3.40S |
| RU | 24.02S | 2.77S | 2.11S | 10.10S | 46.12S | 6.71S |
