# Supplementary material for: Routine laboratory biomarker signature combined with machine learning for rapid differentiation of intracerebral hemorrhage from acute ischemic stroke
Source: Front Neurol. 2026 Oct 15;17:1931764. doi: 10.3389/fneur.2026.1931764 (PMC13716555; doi:10.3389/fneur.2026.1931764)
Supplement: Supplementary file 2 [file Data_Sheet_2.ZIP › Supplemental Table 7-10.docx]

**Supplementary Table 7. Baseline characteristics of temporal validation and Control cohorts**

|  | Control (n = 2893) | ICH (n = 153) | IS (n = 342) |
| --- | --- | --- | --- |
| Age, years | 59(50, 71) | 62(53, 69) | 64(54, 72) |
| Sex |  |  |  |
| Male | 1778 | 105 | 259 |
| Female | 1115 | 48 | 83 |
| Neutrophil Ratio (NEUT%), % | 0.586(0.532, 0.65) | 0.819(0.721, 0.882) | 0.635(0.565, 0.704) |
| White Blood Cell Count (WBC), ×10E9/L | 5.81(4.83, 6.89) | 9.02(6.83, 12.74) | 6.485(5.3, 7.978) |
| Total Protein (TP), g/L | 68.2(64.2, 72.3) | 68.6(63.8, 74.4) | 66.25(62.4, 70.8) |
| Uric Acid (UA), μmol/L | 324(266, 388) | 265(209, 362) | 308.5(252.5, 359.75) |
| Cystatin C (CysC), mg/L | 0.97(0.86, 1.13) | 0.86(0.74, 1.07) | 1.01(0.873, 1.198) |
| Potassium (K^+^), mmol/L | 3.96(3.71, 4.19) | 3.79(3.46, 4.08) | 3.98(3.73, 4.198) |
| Sodium (Na^+^), mmol/L | 143.3(141.4, 145) | 140.1(136.3, 144.1) | 142.75(140.4, 145.175) |
| Chloride (Cl^-^), mmol/L | 105(102.8, 106.9) | 104.6(101.2, 108.2) | 105.7(103.225, 107.775) |
| Fibrinogen Degradation Products (FDP), μg/ml | 1.43(1.03, 2.04) | 2.32(1.05, 6.48) | 1.005(0.663, 1.898) |
| D-Dimer, mg/mL | 0.23(0.19, 0.42) | 0.54(0.26, 1.54) | 0.23(0.13, 0.5) |

**Supplementary Table 8. Temporal validation results without resampling**

|  | **SEN** | **SPE** | **ACC** | **PPV** | **NPV** | **F1** | **AUC** |
| --- | --- | --- | --- | --- | --- | --- | --- |
| **without resampling** | 89.47% | 65.16% | 81.96% | 85.17% | 73.50% | 87.27% | 0.861 |

**Supplementary Table 9. Performance of the model in differentiating between IS patients and controls**

|  | **IS vs Control** | | **ICH vs Control** | |
| --- | --- | --- | --- | --- |
|  | **Training cohort** | **Test cohort** | **Training cohort** | **Test cohort** |
| SEN | 97.21% | 85.6% | 98.23% | 92.52% |
| SPE | 94.42% | 82.81% | 99.75% | 96.21% |
| ACC | 95.81% | 84.21% | 98.99% | 94.36% |
| PPV | 94.57% | 83.28% | 99.75% | 96.06% |
| NPV | 97.13% | 85.19% | 98.26% | 92.79% |
| AUC | 0.989 | 0.923 | 0.995 | 0.979 |

**Supplementary Table 10. Genetic correlations between exposures and stroke outcomes**

|  | **ICH** | | | **IS** | | |
| --- | --- | --- | --- | --- | --- | --- |
|  | **rg** | **rg_se** | **rg_p** | **rg** | **rg_se** | **rg_p** |
| WBC | 0.00234 | 0.06 | 0.969 | -0.133 | 0.165 | 0.422 |
| NEUT% | 0.124 | 0.0594 | 0.037 | -0.0495 | 0.181 | 0.785 |
| CysC | 0.0422 | 0.0544 | 0.438 | 0.273 | 0.171 | 0.11 |
| UA | 0.182 | 0.064 | 0.0045 | 0.383 | 0.189 | 0.0524 |
| TP | 0.0216 | 0.0629 | 0.731 | 0.13 | 0.171 | 0.448 |
| K^+^ | -0.261 | 0.16 | 0.102 | -0.798 | 0.421 | 0.0583 |
| Na^+^ | 0.0279 | 0.0791 | 0.724 | 0.243 | 0.199 | 0.222 |
| Cl^-^ | -0.289 | 0.388 | 0.456 | -0.525 | 0.763 | 0.491 |
| D-Dimer | -0.0621 | 0.324 | 0.848 | 0.202 | 0.893 | 0.821 |
