# Supplementary material for: Routine laboratory biomarker signature combined with machine learning for rapid differentiation of intracerebral hemorrhage from acute ischemic stroke
Source: Front Neurol. 2026 Oct 15;17:1931764. doi: 10.3389/fneur.2026.1931764 (PMC13716555; doi:10.3389/fneur.2026.1931764)
Supplement: Supplementary file 2 [file Data_Sheet_2.ZIP › Supplemental Table 13.docx]

**Supplementary Table 10. Sensitivity Analysis**

| **Exposure** | **Outcome** | **method** | **Q** | **Q_df** | **Q_pval** | **egger_intercept** | **se** | **pval** |
| --- | --- | --- | --- | --- | --- | --- | --- | --- |
| UA | ICH | MR Egger | 252.396 | 255 | 0.534 | 0.006 | 0.003 | 0.065 |
| UA | ICH | IVW | 255.824 | 256 | 0.491 |  |  |  |
| UA | ICH | MR-PRESSO |  |  |  |  |  | 0.492 |
| UA | IS | MR Egger | 138.937 | 123 | 0.154 | 1.476e-05 | 1.454 e-05 | 0.312 |
| UA | IS | IVW | 140.101 | 124 | 0.153 |  |  |  |
| UA | IS | MR-PRESSO |  |  |  |  |  | 0.186 |
| K^+^ | IS | MR Egger | 0.972 | 4 | 0.914 | 0.002 | 0.001 | 0.279 |
| K^+^ | IS | IVW | 2.538 | 5 | 0.771 |  |  |  |
| K^+^ | IS | MR-PRESSO |  |  |  |  |  | 0.796 |
| CysC | IS | MR Egger | 193.806 | 168 | 0.084 | 3.335e-05 | 2.431e-05 | 0.172 |
| CysC | IS | IVW | 195.978 | 169 | 0.076 |  |  |  |
| CysC | IS | MR-PRESSO |  |  |  |  |  | 0.088 |
